# Emergency Braking Evoked Brain Activities during Distracted Driving

**DOI:** 10.3390/s22239564

**Published:** 2022-12-06

**Authors:** Changcheng Shi, Lirong Yan, Jiawen Zhang, Yu Cheng, Fumin Peng, Fuwu Yan

**Affiliations:** 1 Hubei Key Laboratory of Advanced Technology for Automotive Components, Wuhan University of Technology, Wuhan 430070, China; 2 Foshan Xianhu Laboratory of the Advanced Energy Science and Technology Guangdong Laboratory, Foshan 528200, China

**Keywords:** emergency braking, driving distraction, electroencephalogram, statistical parametric mapping, visual distraction, auditory distraction, cognitive distraction

## Abstract

Electroencephalogram (EEG) was used to analyze the mechanisms and differences in brain neural activity of drivers in visual, auditory, and cognitive distracted vs. normal driving emergency braking conditions. A pedestrian intrusion emergency braking stimulus module and three distraction subtasks were designed in a simulated experiment, and 30 subjects participated in the study. The common activated brain regions during emergency braking in different distracted driving states included the inferior temporal gyrus, associated with visual information processing and attention; the left dorsolateral superior frontal gyrus, related to cognitive decision-making; and the postcentral gyrus, supplementary motor area, and paracentral lobule associated with motor control and coordination. When performing emergency braking under different driving distraction states, the brain regions were activated in accordance with the need to process the specific distraction task. Furthermore, the extent and degree of activation of cognitive function-related prefrontal regions increased accordingly with the increasing task complexity. All distractions caused a lag in emergency braking reaction time, with 107.22, 67.15, and 126.38 ms for visual, auditory, and cognitive distractions, respectively. Auditory distraction had the least effect and cognitive distraction the greatest effect on the lag.

## 1. Introduction

Transportation is critical to global economic development and population mobility, but traffic accidents result in many injuries and deaths and cause significant economic losses. According to the National Highway Traffic Safety Administration (NHTSA), 42,915 people died in motor vehicle crashes in the U.S. in 2021, a 10.5% increase from the 38,824 deaths in 2020. Statistics show that almost 95% of motor vehicle crashes are due in some way to driver behaviors, and one main cause of crashes is the failure to take necessary action immediately in an emergency [1]. Therefore, effective detection of the driver’s braking intention earlier can reduce accidents and mitigate the extent of injuries.

Some researchers use onboard sensors to detect potential crashes [2,3,4,5,6,7], and others detect the emergency braking of drivers by using their behavioral data [8,9,10,11,12,13]. Considering that observable behavioral actions are preceded by brain activity, some researchers use driver’s electroencephalogram (EEG) signals to detect emergency braking intentions [14,15]. Haufe et al. [14] were the first to use a driver’s EEG and leg electromyogram (EMG) to detect emergency braking intentions based on a driving simulation system and compared with pedal response. The simulated assisting system using EEG and EMG detected emergency braking 130 ms earlier than the system relying on pedal response only, with a detection accuracy of more than 95%. Subsequently, this experiment was replicated in a real car on a non-public test track, achieving similar results to the driving simulation [16], which fully demonstrates the feasibility and effectiveness of EEG in detecting emergency braking intent. Since then, several studies have extended and refined this, such as rising from emergency braking to soft braking and sudden braking [15] and extending from front vehicle deceleration to a total of three emergency situations (e.g., front vehicle deceleration, other vehicle cut-in, and pedestrian intrusion) [17].

Currently, the research on EEG-based emergency braking intention detection and recognition mainly focuses on EEG feature extraction and classification algorithms. In terms of feature extraction, some researchers select a particular band or combine multiple bands of EEG signals [18,19,20] or select event-related potential (ERP), readiness potential (RP), and event-related desynchronization (ERD) features of EEG signals [15,21,22], and others choose neural correlation features of the brain [1,23]. In terms of classification methods, some researchers use traditional machine learning algorithms [18,19,20,21,22], such as linear discriminant analysis (LDA) and support vector machines (SVM) classification algorithms, which are widely used in offline and online EEG classification, especially online EEG classification [24], while others adopt deep learning [25,26] or combine machine learning with deep learning [27,28], all of which achieve better results.

The above EEG-based emergency braking studies mainly focus on selecting EEG features and classification algorithms to achieve fast and accurate detection of the driver’s emergency braking intention. However, the neural activity mechanism of the driver’s brain during emergency braking is less studied. In addition, the driving environments studied above are fully controlled and ignore the effects of the driver’s state (e.g., stress, fatigue, and distraction) on emergency braking [29,30]. The attention levels during driving tasks are influenced by stress, distraction, and fatigue, and therefore, drivers tend to increase braking reaction times [31,32,33,34]. In recent years, with the application of neurocognitive methods in driving safety research, a few neuroimaging studies have used driving simulators to reveal brain neural activity during driving [35,36,37,38,39,40,41,42,43]. As research has progressed, some researchers use neuroimaging based on driving simulators to explore brain neural activity during distracted driving [44,45,46,47,48], including visual distraction [44,49,50], auditory distraction [45,46,47,51,52,53], and cognitive distraction [42,48]. These studies further reveal the neurological brain mechanisms underlying distracted driving.

Therefore, studying the brain activity mechanism of drivers during emergency braking in different distraction states can improve the diversity and accuracy of EEG feature selection and help detect the driver’s emergency braking intention quickly and accurately. We hypothesize that emergency braking is related to the human attentional state, and the brain activity characteristics and external driving behavior of emergency braking in a distraction state will show some different features. To test this hypothesis, we built a complex traffic scene and designed three distracted subtask paradigms (visual, auditory, cognitive distraction) and a pedestrian intrusion emergency braking stimulus module on Unity3D and further conducted distracted driving emergency braking experiments based on a Logitech G29 driving simulator. EEG signals and the braking reaction time of drivers were collected simultaneously during the experiments and used to investigate the effects of different distractions on the neural mechanism and braking time of the driver during emergency braking. The results show that the common brain regions activated during emergency braking in different distracted states include the inferior temporal gyrus, associated with visual information processing and attention; the left dorsolateral superior frontal gyrus, related to cognitive decision making; and the postcentral gyrus, supplementary motor, and paracentral lobule, associated with motor control and coordination. As the complexity of the task is raised, the extent and degree of activation in prefrontal regions related to cognitive function and prolongation of braking response time increase accordingly.

## 2. Materials and Methods

### 2.1. Experiments

#### 2.1.1. Subjects

In total, 42 subjects were recruited. Their visual acuity, color vision, hearing, and neurological functions were examined before the experiments, and those who passed the examinations could participate in the experiments. They were informed about the experiments three days in advance and practiced for two hours before the formal experiments to familiarize themselves with the equipment and the experiments, and those who met the requirements could participate in the formal experiments. After screening, 12 subjects did not meet the requirements and were eliminated. Thirty subjects (15 males and 15 females) who met the requirements were enrolled, and their ages ranged from 23 to 31 years old (mean = 26.6; SD = 0.9). They all had driver’s licenses, and their driving experience ranged from 1.5 to 9.5 years, with a mean of 4.6 years. They were asked to stop drinking alcohol and coffee for 12 h before the experiments and sleep well. All subjects gave written informed consent before the experiments, indicating they voluntarily participated in the study and received some payment. This study and all its procedures were reviewed and approved by the Ethics Committee of the Wuhan University of Technology.

#### 2.1.2. Experimental Device

**Figure 1:**
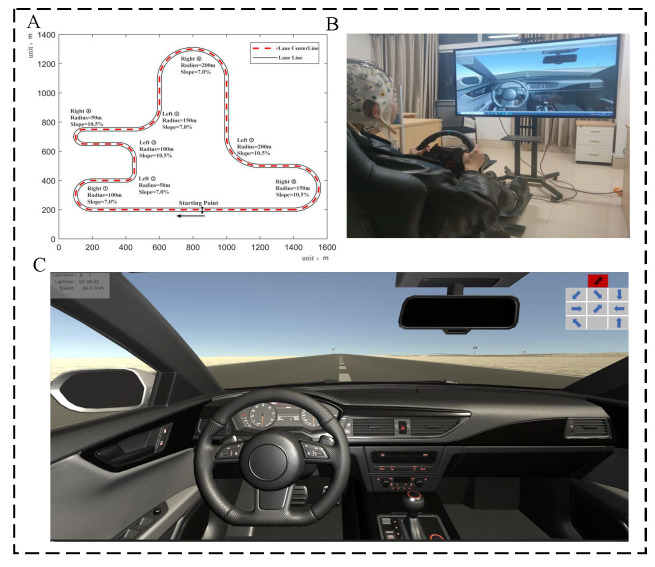
Simulated driving platform. (**A**) The road for the simulation was designed with curves and slopes to simulate a real driving road. (**B**) For the simulated scenario, the subject wore an EEG cap on the head, and EEG information was collected while simulating driving. (**C**) Simulation of the driving display screen showed the current lap number and speed in the upper left corner, and the visual and cognitive distraction screen in the upper right corner when visual or cognitive distraction occurred.

This experiment was designed on Unity3D and Logitech G29 to build a driving simulation system that can collect driving performance data and EEG data simultaneously (Figure 1). The EEG acquisition equipment uses BP’s 64-channel EEG product, ActiCHamp (BRAIN PRODUCTS, Germany, https://www.brainproducts.com/solutions/actichamp/, accessed on 3 August 2020), to collect EEG data from the drivers in real time. The experimental scene was built on Unity3D and displayed by a 55-inch monitor. To simulate the natural road conditions as much as possible, we designed the test road as a single lane with curves and slopes in both directions, separated by white lane lines. Each lane is 3.75 m wide, and the total length of the road is 5.4 km. The road consists of 8 curves with radii of 100, 50, 100, 50, 150, 200, 200, and 150 m, respectively. Each curve is a different turn, and is a linear combination of different turning radii, slopes, and steering. The slope is 4° or 6°. The curve steering is a symmetrical design, and each curve is equipped with a turn sign before. The experimental road is a closed-loop road with no traffic lights, bifurcation, pedestrians or other vehicles. During the experiments, the display showed the current number of laps, the current speed, and the offset from the road centerline in real-time, giving feedback to the driver to maintain the speed and control the vehicle so that it travelled along the centerline.

#### 2.1.3. Experimental Paradigm

The experiment was divided into two parts: a 3-lap normal driving emergency braking experiment, and a 7-lap distracted driving emergency braking experiment. A total of one pedestrian intrusion emergency braking stimulus module and three distraction subtasks were designed: visual distraction, auditory distraction, and cognitive distraction. In the normal driving emergency braking experiment, the driver drove the vehicle normally, while the pedestrian intrusion emergency braking stimulus appeared at any time, with a maximum of 12 stimuli per lap, so approximately 3 × 12 × 30 = 1080 normal driving emergency braking samples could be obtained. In the distracted driving emergency braking experiment, the driver drove the vehicle for 7 laps while the three distracting subtasks appeared randomly, and the pedestrian intrusion emergency braking stimulus appeared at any time during the driver’s completion of the distracting subtasks. Approximately 7 × 12 × 30 = 2520 distracted driving emergency braking samples could be obtained, with an average of approximately 840 samples for each distracted emergency braking, which satisfied the experimental requirements. Both experiments required the driver to drive at a constant speed along the centerline of this lane on the simulator for approximately 5 min per lap, with a maximum speed limit of 90 km/h. The number of subtasks per lap was determined after several preliminary experiments. We found if too few subtasks per lap were performed, the experiment took a much longer time to acquire enough samples, which resulted in fatigue and intolerance according to the post-experiment report of the subjects. If too many subtasks were performed per lap, however, the interval between the subtasks was too short while maintaining the vehicle speed at 90 km/h. The subject did not have enough time to return to the normal driving state before the next distraction subtask, resulting in a baseline shift of the EEG signal, which affected the reasonability of the experiment. Finally, according to an analysis of the preliminary experiments, the number of subtasks was set to be 12 per lap.

Pedestrian intrusion emergency braking stimulus module: The pedestrian suddenly appeared at the road edge and quickly ran across the road. The current distance between the pedestrian’s appearance and the vehicle was 2 s time distance at the current vehicle speed (if the vehicle speed is 90 km/h, i.e., 25 m/s, time distance is 2 s × 25 m/s = 50 m). The moment the pedestrian appeared at the road edge was defined as the start of stimulation. Each driver was asked to apply the emergency brake with his/her foot on the brake pedal immediately after spotting the pedestrian.

**Figure 2:**
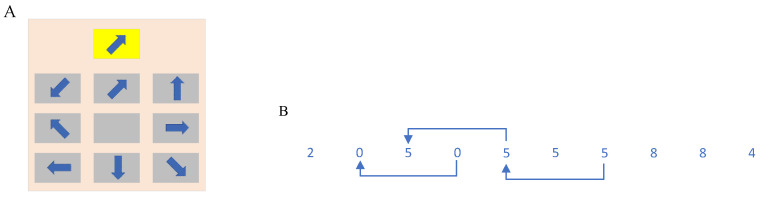
Visual and auditory distraction paradigm. (**A**) Visual distraction paradigm. (**B**) Auditory distraction paradigm.

Visual distraction paradigm: The visual distraction subtask, inspired by the studies of Haste et al. [54] and Liang et al. [55], simulated the visual distraction caused by the driver’s visual attraction to a roadside billboard during driving (Figure 2A). It consisted of one highlighted arrow pattern above and a nine-square consisting of 8 gray arrow patterns in different directions and 1 arrowless space below. These 8 arrow patterns were derived from n rotations, with one rotation of 45°. The highlighted arrow pattern above, and the patterns in the nine-square below changed randomly each time they appeared. The subjects were asked to select the gray arrow pattern in the 3 × 3 nine-pane grid with the same direction as the arrow highlighted above by the left and right paddles under the G29 steering wheel. We coded the nine-square with 3 × 3 matrices, in which the number of times the left paddle and the right paddle were toggled down on G29 indicated the row values and column values, respectively. To select the arrow in row 1 and column 2 in Figure 2a to match the target above, the left toggle was toggled once, and the right toggle was toggled twice. The selected arrow pattern in the nine-square was highlighted, giving the subject feedback on the selection result. The whole process lasted for 5 s, and the system recorded the selection result, the shape of the arrows in each position in the whole picture, and the feedback value of the subject’s paddle to judge whether the subject’s selection was correct. If the paddle feedback task was completed within 5 s, the trial was considered valid whether the feedback was correct or not. Otherwise, the trial was considered invalid, and the invalid trial data were eliminated from the subsequent data processing.

Auditory distraction paradigm: The auditory distraction subtask was drawn on the n-back memory paradigm to model the auditory distraction caused by a driver answering a phone call or talking to a passenger while driving. This paradigm was both an auditory distraction subtask and a simple cognitive distraction subtask to better fit the reality of talking on the phone or with passengers while driving. In this experiment, a random number series consisting of 10 digits from 0–9 was broadcast by voice. Each digit was broadcast for 1.5 s, and the total time of one auditory distraction subtask was 15 s. The 2-back paradigm was used (Figure 2B). During the speech announcement, if the 0–9 digits announced at moment x(n) were heard to be the same as the digits announced at moment x(n−2), the right paddle under the steering wheel need to be pressed to feedback the task result. If not, no paddle feedback was required. During this process, the vehicle was in motion. The system will record each number series and the result of the subject’s paddle feedback to determine whether the subject’s selection was correct.

Cognitive distraction paradigm: The cognitive distraction task was based on the arrow pattern in Figure 2A, and was combined with a voice announcement prompt to rotate clockwise or counterclockwise n times (n = 1, 2, 3, 4, each rotation 45°) the arrow highlighted at the top (e.g., the voice announcement prompted “rotate clockwise 3 times”). From the 8 arrow patterns in the nine-square below the pattern, the subject was prompted by the voice announcement to select the same arrow as the arrow highlighted at the top of the nine-square after n rotations and to use the left and right paddles under the steering wheel to encode the feedback. The numbers of times the left paddle and the right paddle were pushed down indicate the row value and column value respectively. The system lighted up the arrows selected by the left and right paddles in the nine-square, and the whole process lasted for 10 s. The system will record the arrow shape, voice prompt, and paddle feedback value for each position in the nine-square to determine whether the subject’s selection was correct. If the subject did not complete the toggle feedback within 10 s, the trial was considered invalid, and the invalid trial data were eliminated from the subsequent data processing.

### 2.2. Data Acquisition and Preprocessing

The EEG signals of the subjects were acquired using the 64-channel EEG product ActiCHamp. Before the acquisition, the contact impedance between the EEG electrodes and the cortex was calibrated to be less than 5 kΩ. The acquired EEG data were filtered using a low-pass filter and a high-pass filter with cutoff frequency of 50 and 0.1 Hz, respectively, to eliminate DC voltage and high-frequency artifacts, and the average value of the mastoid electrodes was re-referenced. The EEG signals were marked according to the mark information recorded at the time of ActiCHamp acquisition and to the event information recorded by Unit3D. The emergency braking occurred during different distractor subtasks at different marks. The EEG data of the normal driving state, except for the distractor subtask and the emergency braking stimulus, was marked once per second in seconds. The EEG data of the emergency braking state was taken as one trial from the beginning of the mark, i.e., 0 ms to 600 ms after the mark. Five types of trial data (normal driving; emergency braking in normal driving, in visual distraction driving, in auditory distraction driving, in cognitive distraction driving) for each subject were imported into the SPM12 toolkit (Wellcome Department of Imaging Neuro-science, London, UK, Wellcome Trust Centre for Neuroimaging; http://www.fil.ion.ucl.ac.uk/spm/, accessed on 3 August 2020) of Matlab R2021b (MathWorks, Torrance, CA, USA) separately. A series of pre-processing tasks, such as format conversion, montage, high-pass and low-pass filtering, and artifact removal were performed with the data. For example, 0.1 to 45 Hz filtering was performed using Butterworth to remove fractions greater than 100 µV and reduce interference from oculomotor and muscle activity.

To study the effects of different distracted driving patterns on the emergency braking reaction time, we collected the emergency braking stimulus module appearance time and the first time of brake pedal deflection at each trial. This time difference was the driver’s reaction time to emergency braking by pressing the brake pedal with his foot.

### 2.3. Data Processing

A 3D source reconstruction of the preprocessed EEG data was performed to map the 2D sensor data into the 3D brain space and to obtain the precise localization of brain activity. Source space modeling, data alignment, forward computation using the boundary element method, and inverse source reconstruction using multiple sparse prior algorithms were performed. Based on the classic Bayesian approach, the time windows were set from −100 to 1000 ms for normal driving data and from −100 to 600 ms for data of visual, auditory, and cognitive distraction and normal driving emergency braking. Finally, 3D images were obtained from each subject in each trial of the five driving conditions. With the five driving conditions as independent variables, subjects and gender as covariates, and the 3D source reconstructed images as dependent variables, analysis of variance (ANOVA) was performed using SPM to obtain brain area activation results and to analyze the differences in brain area activation during visual, auditory, and cognitive distracted driving and emergency braking in normal driving.

We chose emergency braking reaction time to analyze the effect of distracted driving on emergency braking. ANOVA was performed with the data of emergency braking reaction time from the 30 subjects for three distracted driving states and the normal driving state using SPSS.24 (IBM, Armonk, NY, USA, https://www.ibm.com/support/pages/downloading-ibm-spss-statistics-24, accessed on 3 August 2020) (*p* < 0.05). Post-hoc multiple comparisons were performed using the LSD.

## 3. Results

### 3.1. Emergency Braking Activates Brain Areas in Four Driving Conditions

**Figure 3:**
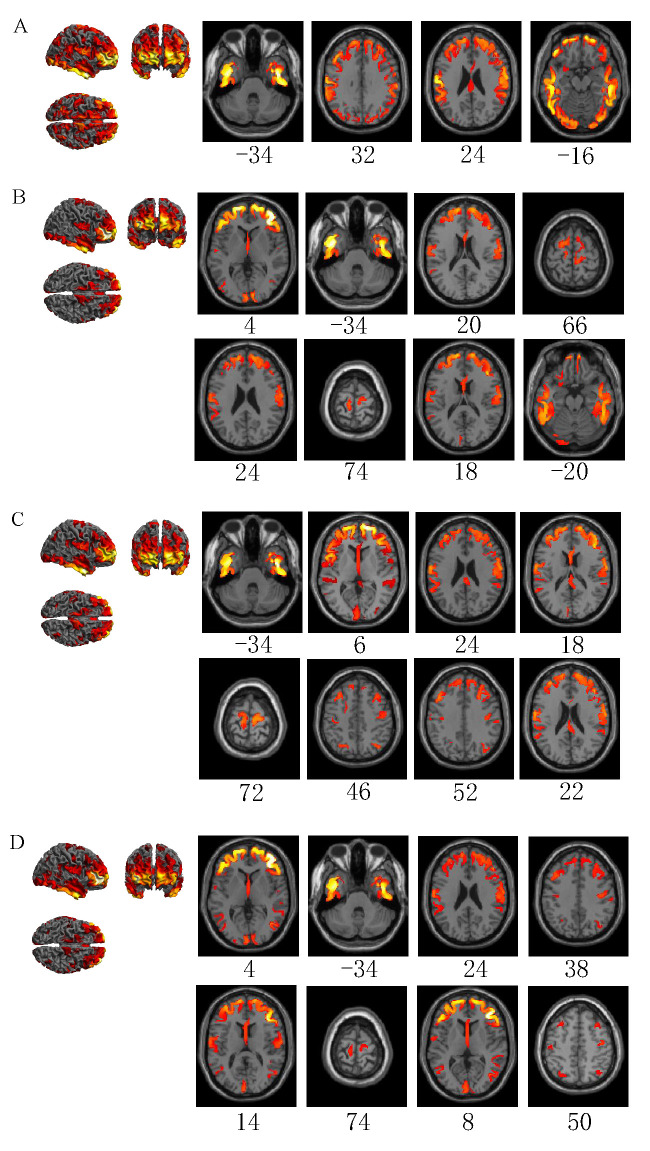
Emergency braking brain area activation in four driving conditions (*p* < 0.05(FWE), extent threshold k > 100 voxels). (**A**) Normal driving. (**B**) Visual distraction driving. (**C**) Auditory distraction driving. (**D**) Cognitive distraction driving. Below the axial-viewed image is the Montreal Neurological Institute (MNI) Z-coordinate of the peak of the current activation cluster.

In total, 2910 sets of 3D source reconstructed images were obtained from the 30 subjects upon emergency braking under four driving conditions. The activation results under each condition are shown in Figure 3 and Table 1 (*p* < 0.05, FWE-corrected, extent threshold k > 100 voxels). The name of the area is derived from the AAL (Anatomical Automatic Labeling, MNI, Montreal, QC, Canada).

The brain regions activated by emergency braking under normal driving are the right inferior temporal gyrus, left angular gyrus, left posterior cingulate gyrus, and left superior temporal gyrus (Figure 3A). The brain regions activated by emergency braking under visual distraction include the right middle frontal gyrus, left and right inferior temporal gyri, left dorsolateral superior frontal gyrus, left and right postcentral gyri, left supplementary motor, left and right paracentral lobules, left supramarginal gyrus, and left inferior frontal gyrus in the orbit (Figure 3B). The brain regions activated by emergency braking under auditory distraction are the left and right inferior temporal gyri, left dorsolateral superior frontal gyrus, right medial superior frontal gyrus, left and right postcentral gyri, right supplementary motor, left paracentral lobule, right angular gyrus, left superior parietal gyrus, and right superior temporal gyrus (Figure 3C). The brain regions activated by emergency braking under cognitive distraction include the right middle frontal gyrus, left and right inferior temporal gyri, left dorsolateral superior frontal gyrus, left and right postcentral gyri, right supramarginal gyrus, left and right paracentral lobules, left middle temporal gyrus, left superior parietal gyrus, right supplementary motor, and right angular gyrus (Figure 3D).

### 3.2. Differences in Brain Area Activation between Distracted Driving Emergency Braking and Normal Driving

**Figure 4:**
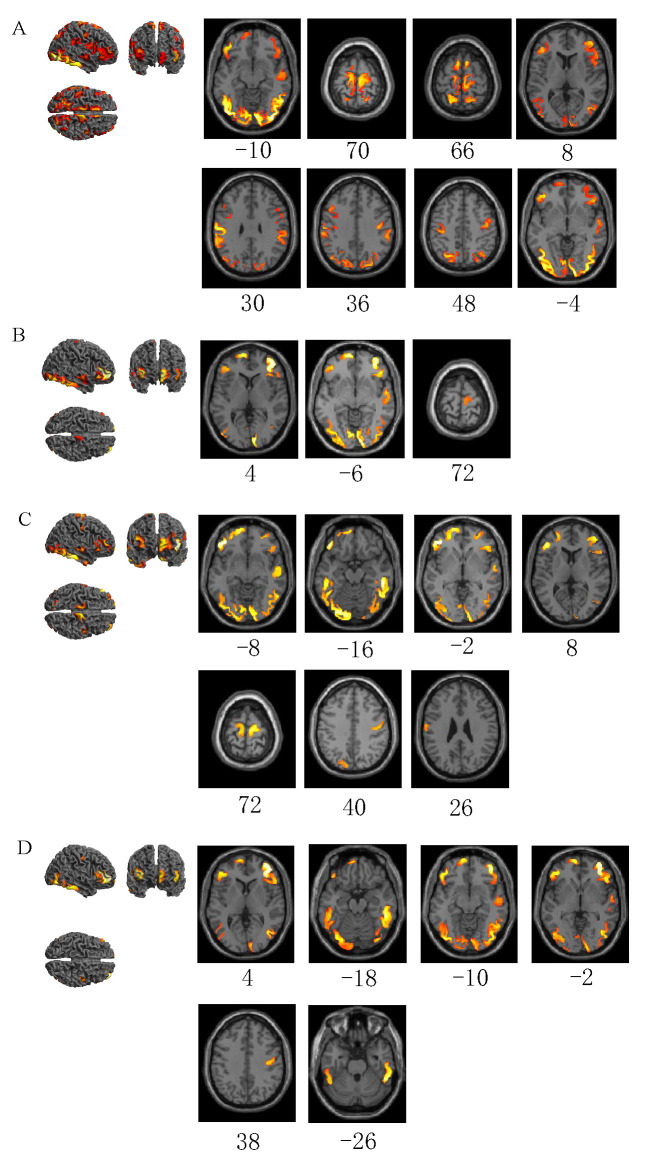
ANOVA of emergency braking in four driving conditions vs. normal driving (*p* < 0.001(FWE), extent threshold k > 100 voxels). (**A**) Emergency braking under normal driving vs. normal driving. (**B**) Emergency braking under visual distraction driving vs. normal driving. (**C**) Emergency braking under auditory distraction driving vs. normal driving. (**D**) Emergency braking under cognitive distraction driving vs. normal driving. Below the axial-viewed image is the Montreal Neurological Institute (MNI) Z-coordinate of the peak of the current activation cluster.

The differences between visual, auditory, or cognitive distracted driving emergency braking and normal driving (*p* < 0.001, FWE-corrected, extent threshold k > 100 voxels) are shown in Figure 4 and Table 2.

The brain areas activated by emergency braking in normal driving–normal driving are the left inferior occipital gyrus, right paracentral lobule, left and right supplementary motors, left inferior frontal gyrus in the orbit, left superior parietal gyrus, right middle frontal gyrus, right supramarginal gyrus, left and right postcentral gyri, right superior temporal gyrus, left middle frontal gyrus, and left superior frontal gyrus in the orbit (Figure 4A). The brain regions activated by emergency braking–normal driving in visual distraction include the left and right lingual gyri, right middle frontal gyrus, left middle frontal gyrus in the orbit, right superior temporal gyrus, left inferior frontal gyrus in the orbit, and right supplementary motor (Figure 4B). The brain regions activated by emergency braking–normal driving in auditory distraction are the left inferior frontal gyrus in the orbit, right inferior temporal gyrus, left lingual gyrus, right calcarine fissure and surrounding cortex, left middle frontal gyrus in the orbit, right middle frontal gyrus, right supplementary motor, right triangular inferior frontal gyrus, right superior temporal gyrus, left paracentral lobule, left and right postcentral gyri, and left cuneus (Figure 4C). The brain areas activated by emergency braking–normal driving in cognitive distraction are the right middle frontal gyrus, left and right inferior temporal gyri, left inferior occipital gyrus, left and right inferior frontal gyri in the orbit, left middle frontal gyrus in the orbit, right calcarine fissure and surrounding cortex, and right postcentral gyrus (Figure 4D).

### 3.3. Differences in Brain Area Activation between Distracted and Normal Driving Emergency Braking

**Figure 5:**
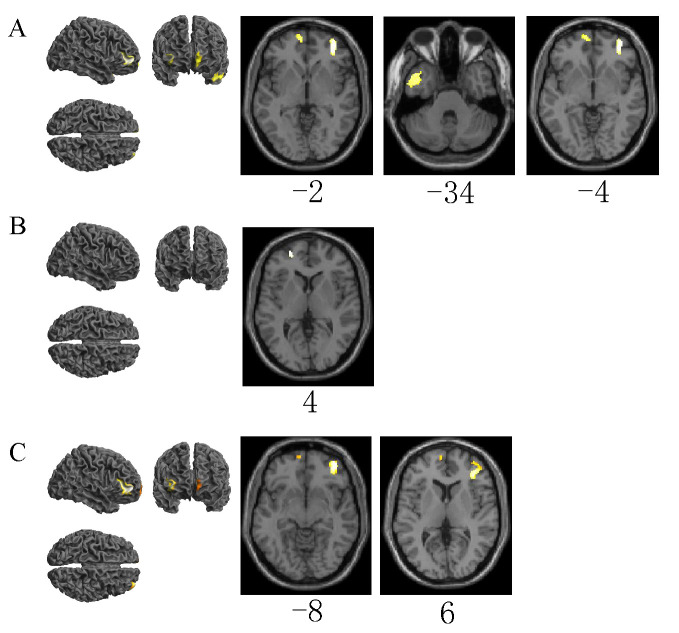
ANOVA of emergency braking in distracted driving vs. emergency braking in normal driving (*p* < 0.001(FWE), extent threshold k > 50 voxels). (**A**) Visual distraction driving vs. normal driving. (**B**) Auditory distraction driving vs. normal driving. (**C**) Cognitive distraction driving vs. normal driving. Below the axial-viewed image is the Montreal Neurological Institute (MNI) Z-coordinate of the peak of the current activation cluster.

The differences in emergency braking between visual, auditory, or cognitive distracted driving and normal driving (*p* < 0.001, FWE-corrected, k > 100 voxels) are shown in Figure 5 and Table 3.

The brain regions activated in emergency braking in visual, auditory, or cognitive distraction–emergency braking in normal driving are the left and right middle frontal gyri in the orbit, left inferior temporal gyrus; the left dorsolateral superior frontal gyrus; the right middle frontal gyrus in the orbit, and the left medial superior frontal gyrus, respectively (Figure 5A–C).

### 3.4. Braking Response Time Differences between Distracted and Normal Driving Emergency Braking

**Figure 6:**
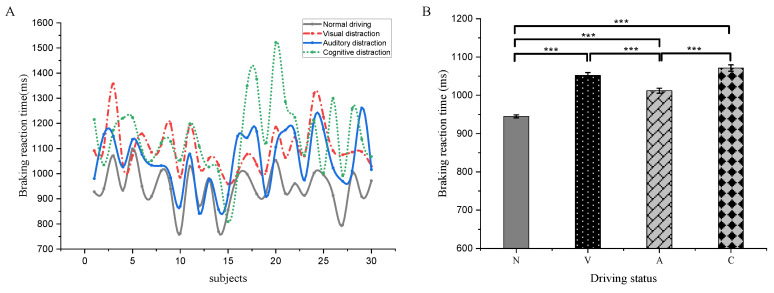
Analysis of emergency braking response time under four driving states. (**A**) Average response time. (**B**) Statistical differences in response time. (*** *p* < 0.001).

In total, 2910 sets of emergency braking reaction time data were collected, of which 1259, 468, 537, and 646 sets were from normal, visual, auditory, and cognitive distraction emergency braking, respectively. The results of average emergency braking reaction time from the 30 subjects in normal driving, visual distraction, auditory distraction, and cognitive distraction are 944.86 ± 3.96, 1052.08 ± 7.57, 1012.01 ± 6.44, and 1071.24 ± 8.35 ms, respectively (Figure 6A). The emergency braking reaction time is the shortest under normal driving condition and the longest under cognitive distracted driving condition. One-way ANOVA (*p* < 0.05) reveals the emergency braking reaction time is significantly different among the four driving conditions (F = 104.385, *p* < 0.001), except for the insignificant difference between visual distraction and cognitive distraction (Figure 6B).

## 4. Discussion

### 4.1. Emergency Braking Activates Brain Areas in Normal Driving Condition

Results showed that emergency braking in normal driving had four activation clusters that survived after correction (*p* < 0.05, k > 100 voxels). The largest cluster in the MNI system peaked in activation at the right inferior temporal gyrus ([46 −4 −34], T = 24.71). The other three clusters were in the left angular gyrus, left superior temporal gyrus, and left posterior cingulate gyrus. The most significant activation was in the right inferior temporal gyrus, with no significant activation in the occipital lobe, which is responsible for visual perception. This result suggests the brain recruits a large number of resources to process visual information at the expense of the occipital regions of visual perception when an emergency arises.

When an emergency occurs, the driver’s primary visual attention shifts from the roadway to the intruding pedestrian, recognizing him or her and making an emergency braking decision. The right inferior temporal gyrus and the left superior temporal gyrus are activated, probably to recognize pedestrians. The inferior temporal gyrus primarily processes complex visual information [56], such as recognizing objects, locations, faces, and colors [57,58]. The superior temporal gyrus mainly recognizes spatial information related to visual objects. Activation of the left angular gyrus and left posterior cingulate gyrus may be associated with attention shifting, spatial processing, and decision-making. Previous studies with functional magnetic resonance imaging (fMRI) show that the angular gyrus is closely related to attentional mechanisms [59,60]. The posterior cingulate cortex is involved in spatial processing, spatial actions, and certain types of memory [61,62]. The posterior cingulate gyrus is also extensively linked to memory, attention, and decision-making [63,64,65,66]. Other evidence suggests that the posterior cingulate gyrus plays a more direct role in regulating attentional focus [67,68,69].

Emergency braking vs. normal driving ANOVA showed that occipital, temporal, and parietal regions, associated with visual perception, information processing, and spatial perception, were activated during emergency braking compared to normal driving, such as the left inferior occipital gyrus, left superior parietal gyrus, right superior temporal gyrus, and right superior limbic gyrus. In particular, the left inferior occipital gyrus was significantly activated. Frontoparietal lobes associated with cognitive decision-making were also activated, such as the left inferior orbital frontal gyrus, left and right middle frontal gyri, left superior orbital frontal gyrus, and left superior parietal gyrus. The posterior parietal cortex associated with motor control and planning was also significantly activated, such as the right paracentral lobule, supplementary motor, postcentral gyrus, and right supramarginal gyrus. The activation of these three functional areas corresponds to the three components of the driver’s disposition of emergency braking situations, including emergency state perception and recognition, cognition and decision-making, and motor control and coordination.

Previous studies with functional near-infrared spectroscopy (fNIRS) show that the cortical postcentral gyrus, primary visual cortex V1, and limbic supramarginal gyrus are more active during emergency braking before the driver depresses the brake pedal than during constant speed driving. The activation of the primary visual cortex V1 indicates the driver pays attention to road information. An active supramarginal gyrus suggests the driver is spatially oriented to the motion of the foot and brake pedal. An active postcentral gyrus implies the driver seeks the brake pedal and that the information integration function of the postcentral gyrus also plays an important role [70]. Our results are in general agreement with previous studies. The difference is that our experiment activates a wider area, which may be due to the experimental equipment and our complex experimental scenario with many curves and hills.

### 4.2. Emergency Braking Neural Activation in Visual Distraction

The results showed that temporal lobes related to visual information processing (e.g., inferior temporal gyrus, left superior limbic gyrus), frontal lobes related to cognitive decision making (e.g., right middle frontal gyrus, left dorsolateral superior frontal gyrus, left inferior orbital frontal gyrus), and posterior parietal lobes related to motor control and coordination (e.g., postcentral gyrus, supplementary motor area, paracentral lobule) were significantly activated during emergency braking in visual distraction. The visual information processing and cognitive decision-making were more activated during visually distracted emergency braking. The driver need to process visual information and make decisions when completing the visual distraction task, which, in addition to dealing with the emergency of pedestrian intrusion, required the processing of abundant visual information and timely decision-making. This may explain the high activation of these two functional areas. The right middle frontal gyrus is considered to be the convergence point of the dorsal and ventral attentional network and plays an important role in redirecting attention from exogenous to endogenous attentional control [71]. This gyrus is active only when redirected to unexpected stimuli [72,73]. The dorsolateral prefrontal cortex has important executive functions, such as working memory, cognitive flexibility [74], planning, inhibition, and abstract reasoning [75]. This cortex is also the highest cortical area in motor planning, organization, and regulation [76].

The ANOVA showed that during visual distraction emergency braking vs. normal driving, the driver’s main focus was on perceiving and recognizing the emergency and on making decisions based on the situation. As a result, the lingual gyrus and right superior temporal gyrus associated with visual information processing, the right middle frontal gyrus, left intraorbital superior frontal gyrus, and left infraorbital frontal gyrus related to cognitive decision-making, and the supplementary motor associated with the movement were all significantly activated. The lingual gyrus is more active during visual processing [77]. The right superior temporal gyrus serves as a convergence point between dorsal and ventral visual streams and contributes to the processing of object- and space-related information [78]. Moreover, spatial awareness in humans is largely limited to the function of the right superior temporal cortex [79].

Results showed that the load on visual processing and cognitive decision-making was greater during emergency braking with visual distraction vs. normal driving. Thus, the right inferior temporal gyrus associated with visual processing and the left orbital middle frontal gyrus and left intraorbital superior frontal gyrus associated with cognitive decision-making were significantly activated. These results are consistent with our hypothesis.

### 4.3. Emergency Braking Neural Activation in Auditory Distraction

Emergency braking in auditory distraction driving ANOVA showed that the inferior temporal gyrus, right angular gyrus, and left parietal gyrus associated with visual information processing; the left dorsolateral superior frontal gyrus and right medial superior frontal gyrus related to cognitive decision-making; the postcentral gyrus, right supplementary motor, and left paracentral lobule associated with motor control; the right superior temporal gyrus and right angular gyrus related to hearing were more active. Among them, brain areas related to visual processing and cognitive decision-making were significantly activated. These results can be explained by the fact that the emergency braking task is more important compared to the auditory distraction task, which requires the recruitment of brain resources for the recognition of emergencies. Furthermore, the 2-back paradigm used for the auditory distraction task is both an auditory and a simple cognitive task involving memory. Emergency braking during the completion of a 2-back paradigm task can significantly increase cognitive load. Moreover, the auditory and prefrontal regions are anatomically and functionally interconnected [80,81], the prefrontal cortex is involved in auditory attention tasks [82], and the prefrontal lobe is also closely related to working memory [83].

The ANOVA of emergency braking in auditory distraction driving vs. normal driving showed that activation was mainly distributed in the right inferior temporal gyrus, left lingual gyrus, right perirhinal cortex, and left cuneus associated with visual information processing, the right superior temporal gyrus related to auditory information processing, the left inferior orbital frontal gyrus, left infraorbital frontal gyrus, right middle frontal gyrus, and inferior deltoid frontal gyrus associated with cognitive decision-making, and the right supplementary motor, the left paracentral lobule, and four regions of the postcentral gyrus related to motor control and coordination. Compared to normal driving, an auditory distraction for emergency braking increased the loads in visual perception and processing (recognizing pedestrian intrusion), auditory perception and processing (2-back task), cognitive decision-making (pedestrian intrusion, 2-back task), and motor control (emergency braking of the vehicle). Regarding the activation level, the driver’s brain areas related to visual information processing and cognitive decision-making were activated to higher degrees during auditory distraction emergency braking, because emergency braking was more important than completing the auditory distraction task.

Results showed that significant activation of the left dorsolateral superior frontal gyrus during auditory distraction emergency braking compared to normal driving emergency braking. This may be because the 2-back paradigm of auditory distraction is a cognitive task involving memory. As reported, the left frontopolar area and the left dorsolateral prefrontal cortex are activated in the n-back task [84]. Frontopolar areas, in particular, play a role in updating memory [85].

### 4.4. Neural Activation of Emergency Braking in Cognitive Distraction

The ANOVA of emergency braking in cognitive distraction driving showed that the right and left inferior temporal gyri, left superior parietal gyrus, and right angular gyrus associated with visual information processing; the right superior limbic gyrus, left middle temporal gyrus, right angular gyrus, and right middle frontal gyrus related to auditory information processing; and the right middle frontal gyrus, left dorsolateral superior frontal gyrus, and left superior parietal gyrus associated with cognitive decision-making were activated. The postcentral gyrus, paracentral lobule, right supplementary motor associated with motor control and coordination and the right supramarginal gyrus were more active. Regarding the degree of activation, brain areas associated with visual information processing and cognitive decision-making were significantly activated. The reasons are that both cognitive distraction and emergency braking tasks require visual processing and cognitive decision-making resources. Reportedly, the human prefrontal cortex is involved in various cognitive processes, including cognitive control, working memory, and attention [86]. In tasks involving sustained attention, the frontal middle gyrus is activated [87]. In addition to the prefrontal cortex, the parietal cortex also participates in cognitive tasks, and any higher-level visual or cognitive task requires activation of the equivalent frontoparietal network in the human brain [88].

Emergency braking in cognitive distraction driving vs. normal driving ANOVA showed that the main activated regions were the inferior temporal gyrus, inferior occipital gyrus, and perirhinal cortex associated with visual information processing; and three regions in the right middle frontal gyrus, left infraorbital frontal gyrus, left intraorbital superior frontal gyrus, right infraorbital frontal gyrus, and postcentral gyrus associated with motor control. The cognitive decision-making functional area was the most severely and widely activated, followed by the visual information processing functional brain area. Compared to normal driving, cognitive distraction emergency braking is equivalent to adding a dual task to normal driving, so the driver needs to handle more dual visual and cognitive tasks.

Emergency braking in cognitive distraction driving vs. normal driving ANOVA showed significant activation in the right orbital middle frontal gyrus and the left medial superior frontal gyrus, of which both are in the frontal lobe. The reason is that cognitive distraction emergency braking requires the driver to recruit more cognitive resources than normal driving emergency braking.

### 4.5. Distracted Driving Emergency Braking Brain Area Activation and Braking Reaction Time

The average emergency braking reaction time of drivers during normal driving, visual distraction, auditory distraction, and cognitive distraction was 944.86 ± 3.96, 1052.08 ± 7.57, 1012.01 ± 6.44, and 1071.24 ± 8.35 ms, respectively. The lagged reaction time was 107.22, 67.15, and 126.38 ms, respectively. Clearly, distracted driving impacts driving safety. In contrast, auditory distraction least affected and cognitive distraction most affected the emergency braking reaction time. This may be related to the increased visual information processing load and cognitive load of the driver during visual distraction and cognitive distraction compared to auditory distraction, and the driver’s visual information processing load increases. Especially during the cognitive distraction, not only the visual information processing load but also the cognitive load increases, the temporal inferior gyrus associated with visual information processing and the prefrontal area associated with cognitive decision-making are significantly activated, and processing emergency braking limits brain resources for the task, leading to a lag in braking response time.

### 4.6. Limitations of the Study

We investigated the mechanisms of emergency braking brain neural activity in visual, auditory, and cognitive distracted driving vs. normal driving, and thereby explored the use of EEG neuroimaging to study the mechanisms of neural activity during emergency braking in different driving states. However, there are some limitations. First, to match the real driving conditions better, we used a road with many curves and hills, which caused the driver to frequently adjust the steering wheel and step on the gas pedal and brake pedal. However, frequent body movements will interfere with the EEG signals, but we cannot completely filter out these artifacts despite using various methods. To solve this problem, in the next step, we will set up control experiments conducted on a straight road without slope or turns, which are worth studying to minimize the motion artifacts. The electrodes in the motion area associated should be carefully analyzed and may be excluded from further analysis. More efficient methods, such as independent component analysis, should be studied to filter out these artifacts. Second, we only chose one kind of emergency braking stimulus, viz., a pedestrian breaking into the road, and did not consider the situation of dogs or other animals breaking into the road, nor did we include two emergency braking stimuli, such as sudden braking of the front car, and cut-in of sidecar. One reason is that pedestrian intrusion is more common and causes more damage on urban roads. Another reason is that other animal intrusions have similar effects on drivers as pedestrian intrusions. This certainly depends on additional research. Further studies will consider the introduction of more types of intrusions and the performance of comparative analyses. Third, the subject was informed before the experiment that an emergency braking stimulus was present during driving, although he/she did not know when the emergency braking stimulus was present, which is somewhat different from the contingency of an emergency braking scenario during actual driving. To address this issue, we took some steps to avoid the effect of such preconceptions on the experimental results. The subjects were randomized as to when and what kind of distraction task would occur during the driving simulation. The emergency braking task was also randomized. Despite that, it is still somewhat different from the chance of emergency braking appearing during actual driving. How to eliminate this effect deserves further study. Finally, to reduce interference to the driver, we chose the driving scenario as a single closed-loop road with no traffic intersections, diversions, or traffic flow, which resulted in a gap between our study and the real-world driving environment. In a follow-up study, a broader population with more subjects, and a large number of experiments on real roads might be helpful to validate and refine our results.

## 5. Conclusions

The main activated brain areas of drivers during emergency braking have three functions: visual information processing, cognitive decision-making, and motor control and coordination. The first two functions are in the higher level than motor control and coordination functions during emergency braking in all distracted driving states. During emergency braking, the driver’s main focus is on recognizing and making decisions about the emergency. The activation level of the corresponding brain regions differed for different distracted driving states of emergency braking. The activation of the prefrontal cortex related to cognitive decision-making was the highest and the activation area was the widest during cognitive distraction emergency braking. All distractions caused a lag in emergency braking reaction time, with 107.22, 67.15, and 126.38 ms for visual, auditory, and cognitive distractions, respectively. The auditory distraction had the least effect and cognitive distraction had the greatest effect on the lag. Remedial measures should be taken to avoid the bad outcomes of these lags. On the one hand, the driver should be more attentive and try to avoid distractions during driving. On the other hand, it is necessary to take relevant technical measures, such as a driving distraction detection method, to detect the real-time attentive state of the driver, and the intervention measures, such as voice warning, to help the driver to recover to the normal state once he/she was found to be distracted.

## Figures and Tables

**Table 1:** Brain activation for emergency braking in four driving conditions.

	MNI-Space Coordinates			k_E_	T	Hemisphere	Region
	x	y	z				
Emergency braking in normal driving	46	−4	−34	39,965	24.71	R	Inferior temporal gyrus
	−48	−70	32	232	8.25	L	Angular gyrus
	0	−32	24	150	5.89	L	Posterior cingulate gyrus
	−30	6	−16	83	4.79	L	Superior temporal gyrus
Emergency braking in visual distraction driving	44	50	4	5360	20.97	R	Middle frontal gyrus
	−44	0	−34	6255	20.74	L	Inferior temporal gyrus
	−16	62	6	4188	19.21	L	Superior frontal gyrus, dorsolateral
	44	−4	−32	5567	17.97	R	Inferior temporal gyrus
	64	−10	20	750	8.66	R	Postcentral gyrus
	−12	2	66	283	7.99	L	Supplementary motor area
	−60	−18	24	543	7.73	L	Postcentral gyrus
	−12	−24	74	206	7.57	L	Paracentral lobule
	8	−22	72	516	7.55	R	Paracentral lobule
	−52	−42	26	164	5.61	L	Supramarginal gyrus
	−22	22	−20	186	5.29	L	Inferior frontal gyrus, orbital part
Emergency braking in auditory distraction driving	46	−4	−34	5446	20.72	R	Inferior temporal gyrus
	−20	62	6	4540	19.01	L	Superior frontal gyrus, dorsolateral
	14	62	6	5376	18.56	R	Superior frontal gyrus, medial
	−44	−2	−34	6045	18.23	L	Inferior temporal gyrus
	−62	−10	24	976	10.17	L	Postcentral gyrus
	64	−8	18	929	10.10	R	Postcentral gyrus
	10	−18	72	621	9.85	R	Supplementary motor area
	−12	−24	74	640	9.14	L	Paracentral lobule
	40	−68	46	245	7.46	R	Angular gyrus
	−28	−68	52	197	6.86	L	Superior parietal gyrus
	62	−40	22	132	5.82	R	Superior temporal gyrus
Emergency braking in cognitive distraction driving	44	50	4	6376	26.65	R	Middle frontal gyrus
	−42	0	−34	6369	23.47	L	Inferior temporal gyrus
	46	−4	−36	6456	22.53	R	Inferior temporal gyrus
	−16	62	4	5164	21.11	L	Superior frontal gyrus, dorsolateral
	−62	−12	24	1002	10.69	L	Postcentral gyrus
	56	−20	26	1219	10.35	R	Supramarginal gyrus
	40	−16	38	403	8.76	R	Postcentral gyrus
	12	−26	68	359	8.13	R	Paracentral lobule
	−12	−26	74	527	7.41	L	Paracentral lobule
	−54	−62	8	201	7.03	L	Middle temporal gyrus
	−30	−68	50	138	6.05	L	Superior parietal gyrus
	40	−68	46	140	5.94	R	Angular gyrus
	10	4	68	107	5.08	R	Supplementary motor area

Note: *p* < 0.05(FWE), extent threshold k > 100 voxels.

**Table 2:** Activation difference in emergency braking vs. normal driving.

	MNI-Space Coordinates			k_E_	T	Hemisphere	Region
	x	y	z				
Emergency braking in normal driving vs. normal driving	−46	−78	−10	9386	12.60	L	Inferior occipital gyrus
	6	−24	70	1689	8.90	R	Paracentral lobule
	−6	10	66	367	8.64	L	Supplementary motor areas
	8	12	64	260	8.60	R	Supplementary motor areas
	−42	38	−14	778	8.37	L	Inferior frontal gyrus, orbital part
	−22	−62	62	1978	8.09	L	Superior parietal gyrus
	44	46	8	1763	7.58	R	Middle frontal gyrus
	52	−32	30	464	6.60	R	Supramarginal gyrus
	40	−16	36	677	6.59	R	Postcentral gyrus
	−36	−24	48	493	6.57	L	Postcentral gyrus
	58	−4	−4	358	6.02	R	Superior temporal gyrus
	−46	14	38	198	5.84	L	Middle frontal gyrus
	−12	58	−16	322	5.31	L	Superior frontal gyrus, orbital part
Emergency braking in visual distraction driving vs. normal driving	8	−84	−10	2205	8.81	R	Lingual gyrus
	44	50	4	1144	8.48	R	Middle frontal gyrus
	−12	64	−2	546	7.89	L	Middle frontal gyrus, orbital part
	−8	−80	−6	2353	7.46	L	Lingual gyrus
	56	−4	−6	353	5.33	R	Superior temporal gyrus
	−40	40	−6	421	5.30	L	Inferior frontal gyrus, orbital part
	8	−20	72	107	4.03	R	Supplementary motor area
Emergency braking in auditory distraction driving vs. normal driving	−40	40	−8	682	8.20	L	Inferior frontal gyrus, orbital part
	52	−36	−16	2411	8.08	R	Inferior temporal gyrus
	−24	−86	−16	2746	7.52	L	Lingual gyrus
	14	−98	−2	421	6.86	R	Calcarine fissure and surrounding cortex
	−14	64	−2	925	6.63	L	Middle frontal gyrus, orbital part
	46	46	8	644	6.47	R	Middle frontal gyrus
	12	−16	72	341	6.40	R	Supplementary motor area
	44	30	0	342	5.86	R	Inferior frontal gyrus, triangular part
	58	−4	−6	309	5.29	R	Superior temporal gyrus
	−10	−24	74	228	5.24	L	Paracentral lobule
	42	−14	36	306	4.85	R	Postcentral gyrus
	−10	−84	40	120	4.29	L	Cuneus
	−62	−10	26	175	4.24	L	Postcentral gyrus
Emergency braking in cognitive distraction driving vs. normal driving	44	50	4	767	11.08	R	Middle frontal gyrus
	52	−38	−18	1258	9.42	R	Inferior temporal gyrus
	−44	−80	−10	420	8.87	L	Inferior occipital gyrus
	−44	34	−16	438	8.48	L	Inferior frontal gyrus, orbital part
	−10	64	−2	256	8.07	L	Middle frontal gyrus, orbital part
	48	32	−2	262	7.77	R	Inferior frontal gyrus, orbital part
	14	−98	−2	142	6.90	R	Calcarine fissure and surrounding cortex
	40	−16	38	198	6.33	R	Postcentral gyrus
	−50	−44	−26	291	6.33	L	Inferior temporal gyrus

Note: *p* < 0.001(FWE), extent threshold k > 100 voxels.

**Table 3:** Activation difference of emergency braking in distraction driving vs. emergency braking in normal driving.

	MNI-Space Coordinates			k_E_	T	Hemisphere	Region
	x	y	z				
Emergency braking (visual distraction driving vs. normal driving)	36	50	−2	384	4.62	R	Middle frontal gyrus, orbital part
	−42	4	−34	290	3.93	L	Inferior temporal gyrus
	−6	60	−4	171	3.85	L	Middle frontal gyrus, orbital part
Emergency braking (auditory distraction driving vs. normal driving)	−22	54	4	52	3.54	L	Superior frontal gyrus, dorsolateral
Emergency braking (cognitive distraction driving vs. normal driving)	40	52	−8	558	5.91	R	Middle frontal gyrus, orbital part
	−6	62	6	89	3.70	L	Superior frontal gyrus, medial

Note: *p* < 0.001(FWE), extent threshold k > 50 voxels.

## Data Availability

The data presented in this study are available on request from the corresponding author.
